# Protective effects of Scutellaria baicalensis Georgi against hydrogen peroxide-induced DNA damage and apoptosis in HaCaT human skin keratinocytes

**DOI:** 10.17179/excli2016-817

**Published:** 2017-03-31

**Authors:** Jung Jeh Yoon, Jin-Woo Jeong, Eun Ok Choi, Min Ju Kim, Hyun Hwang-Bo, Hong Jae Kim, Su Hyun Hong, Cheol Park, Dong Hee Lee, Yung Hyun Choi

**Affiliations:** 1 Anti-Aging Research Center and Department of Biochemistry, Dongeui University College of Korean Medicine, 176 Yangjeong-ro, Busanjin-gu, Busan 47227, Republic of Korea; 2 Lioele Cosmetic Co., LTD., 2068-1 Jungangdae-ro, Geumjeong-gu, Busan 46214, Republic of Korea; 3 Department of Molecular Biology, College of Natural Sciences, Dongeui University, 176 Eomgwangno Busanjin-gu, Busan 47340, Republic of Korea; 4 Genomine Inc., Venture Bldg 306, Pohang TechnoPark, 394 Jigokor Pohang, 37668, Republic of Korea

**Keywords:** Scutellaria baicalensis rhizome, antioxidant, DNA damage, apoptosis, ROS, Nrf2/HO-1

## Abstract

Oxidative stress due to excessive accumulation of reactive oxygen species (ROS) is one of the risk factors for the development of several chronic diseases. In this study, we investigated the protective effects of *Scutellaria baicalensis* rhizome ethanol extract (SBRE) against oxidative stress-induced cellular damage and elucidated the underlying mechanisms in the HaCaT human skin keratinocyte cell line. Our results revealed that treatment with SBRE prior to hydrogen peroxide (H_2_O_2_) exposure significantly increased viability of HaCaT cells. SBRE also effectively attenuated H_2_O_2_-induced comet tail formation and inhibited the H_2_O_2_-induced phosphorylation levels of the histone γH2AX, as well as the number of apoptotic bodies and Annexin V-positive cells. In addition, SBRE exhibited scavenging activity against intracellular ROS generation and restored the mitochondrial membrane potential loss by H_2_O_2_. Moreover, H_2_O_2 _enhanced the cleavage of caspase-3 and degradation of poly (ADP-ribose)-polymerase, a typical substrate protein of activated caspase-3, as well as DNA fragmentation; however, these events were almost totally reversed by pretreatment with SBRE. Furthermore, SBRE increased the levels of heme oxygenase-1 (HO-1), which is a potent antioxidant enzyme, associated with the induction of nuclear factor-erythroid 2-related factor 2 (Nrf2). According to our data, SBRE is able to protect HaCaT cells from H_2_O_2_-induced DNA damage and apoptosis through blocking cellular damage related to oxidative stress through a mechanism that would affect ROS elimination and activating the Nrf2/HO-1 signaling pathway.

## Introduction

Accumulating evidence has demonstrated that excessive exposure to ultraviolet (UV) radiation from sunlight is a major risk factor for skin damage due to the overwhelming generation of reactive oxygen species (ROS) (Kammeyer and Luiten, 2015[21]; Karran and Brem, 2016[22]). Although some ROS physiologically act as cellular messengers in redox signaling, uncontrolled release of ROS is further involved in the pathogenesis of a number of human skin disorders by inducing oxidative DNA damage (Birch-Machin et al., 2013[2]; Katiyar, 2016[24]). Consequently, damaged DNA contributes to the initiation of cellular apoptosis in keratinocytes, and this in turn eventually leads to the disruption of the epithelial structure of the skin (Brunelle and Chandel, 2002[4]; Cadet et al., 2015[5]). Therefore, the development of safer and more effective antioxidants for skin protection continues to be an important research goal.

The accumulation of ROS could modulate the expression of redox-sensitive genes. One of the most important regulators in the defense of cells against oxidative stress is nuclear transcription factor erythroid-2-like factor 2 (Nrf2), a basic leucine zipper transcription factor (Satoh et al., 2013[38]; Loboda et al., 2016[31]). This transcription factor is known to activate the expression of antioxidant response element (ARE)-related expression of phase 2-detoxifying genes and anti-oxidants, such as heme oxygenase-1 (HO-1) and NAD(P)H-quinone oxidoreductase-1 (NQO-1) (Satoh et al., 2013[38]; Loboda et al., 2016[31]). Because these antioxidant enzymes are essential for protecting the keratinocytes from oxidative stress (Gęgotek and Skrzydlewska, 2015[16]; Schäfer and Werner, 2015[39]), increasing the antioxidant capacity of skin cells by antioxidant supplements could be a valuable strategy against oxidative stress-induced skin damage. 

*Scutellaria baicalensis* Georgi is a species of flowering plant in the Lamiaceae family, which is widely distributed in Siberia, Mongolia, the Russian Far East, China, and Korea (Li et al., 2011[29]). The root of *S. baicalensis*, Scutellariae Radix, has been used worldwide in traditional medicine for the treatment of various human diseases (Boyle et al., 2011[3]). Previous studies have indicated that the extracts of *S. baicalensis* and its biogenic active ingredients have various pharmacological properties, including antibacterial (Chung et al., 2014[13]), antioxidant (Lin et al., 2014[30]; Chang et al., 2016[7]), anti-tumor (Choi et al., 2015[9], 2016[11]), anti-obesity (Song et al., 2013[42]), anti-asthmatic (Ko et al., 2014[26]; Zhou et al., 2016[47]), anti-hypotensive (Huang et al., 2005[18]; Deng et al., 2012[15]), neuroprotective (Shang et al., 2013[41]; Moon and Park, 2015[33]), and anti-inflammatory activities (Ku and Bae, 2015[27]; Seok et al., 2016[40]). We have recently demonstrated that the protective effects of baicalein, an active component of *S. baicalensis*, against oxidative stress-induced damage through activation of Nrf2 signaling pathway (Choi et al., 2016[10]). Other researchers have obtained similar results (Zhang et al., 2012[46]; Cui et al., 2015[14]). However, the protective effects and molecular mechanisms involved in oxidative stress-induced skin damages are still unclear. In this study, as a part of our research program to find new antioxidative compounds from the medicinal herbs, we investigated the protective effect of *S. baicalensis* rhizome ethanol extract (SBRE) against hydrogen peroxide (H_2_O_2_)-induced ROS generation, oxidative damage, and apoptosis in HaCaT immortalized non-tumorigenic human keratinocytes. 

## Materials and Methods

### Preparation of SBRE

For the preparation of SBRE, the dried roots of *S. baicalensis* (100 g) were provided by Dongeui Korean Medical Center (Busan, Republic of Korea) and authenticated by Professor S.H. Hong, Department of Biochemistry, Dongeui University College of Korean Medicine. The dried roots (100 g) were cut into small pieces, ground into a fine powder, and then extracted in 1 L of 70 % ethanol by sonication for 3 h. After filtering and concentration of the extracts, the remaining powder was dissolved in dimethyl sulfoxide (DMSO, Sigma-Aldrich Chemical Co., St. Louis, MO, USA) as a stock solution at a 100 mg/ml concentration and stored at 4 °C. The stock solution was diluted with cell culture medium to the desired concentration prior to use.

### Cell culture

HaCaT human skin keratinocyte cell line was obtained from the American Type Culture Collection (Manassas, MD, USA) and cultured in RPMI 1640 medium (WelGENE Inc., Daegu, Republic of Korea) containing 10 % heat-inactivated fetal bovine serum (FBS, WelGENE Inc., Daegu, Republic of Korea), streptomycin (100 μg/mL), and penicillin (100 Units/mL). Cells were maintained at 37 °C in an incubator with a humidified atmosphere of 5 % CO_2_. 

### Cell viability assay

For the cell viability assay, HaCaT cells were seeded in 6-well plate at 2×10^5 ^cells/ml and cultured for 24 h before being treated with various concentrations of SBRE for 24 h in the presence or absence of H_2_O_2 _or N-acetyl-L-cysteine (NAC, Sigma-Aldrich Chemical Co.). After treatments, 3-[4,5-dimethylthiazo l-2-yl]-2,5diphenyltetrazolium bromide (MTT, Sigma-Aldrich Chemical Co., St. Louis, MO, USA) solution (0.5 mg/ml) was added, followed by 2 h-incubation at 37 °C in the dark, and the medium was removed. The formazan precipitate was dissolved in DMSO. The absorbance of the formazan product was measured at 540 nm using an enzyme-linked immunosorbent assay (ELISA) reader (Molecular Devices, Sunnyvale, CA, USA) (Kim et al., 2016[25]). 

### Measurement of intracellular ROS

The oxidation-sensitive dye 2′,7′-dichlorodihydrofluorescein diacetate (DCF-DA, Molecular Probes, Eugene, OR, USA) was used to determine the formation of intracellular ROS. Briefly, the cells were harvested, washed twice with phosphate-buffered saline (PBS), and then re-suspended in 10 μM DCF-DA for 30 min at 37 °C in the dark. ROS production in the cells was monitored immediately by a flow cytometer (Becton Dickinson, San Jose, CA, USA).

### Comet assay (Single-cell gel electrophoresis assay) 

After each treatment, the cells were washed with PBS and the cell suspension was mixed with 0.5 % low melting agarose (LMA) at 37 °C, and added to the slides pre-coated with 1.0 % normal melting agarose. After solidification of the agarose, slides were covered with another 0.5 % LMA, and then immersed in lysis buffer [2.5 M NaCl, 500 mM Na-ethylenediaminetetraacetic acid (EDTA, Sigma-Aldrich Chemical Co.), 1 M Tris buffer, 1 % sodium lauryl sarcosine, and 1 % Triton X-100) for 1 h at 4 °C. Later, the slides were transferred into an unwinding buffer for another 20 min for DNA unwinding. The slides were then placed in an electrophoresis tank containing 300 mM NaOH and 1 mM Na-EDTA (pH 13.0). An electrical field was then applied (300 mA, 25 V) for 20 min at 25 °C to draw the negatively-charged DNA toward the anode. The slides were washed three times for 5 min at 25 °C in a neutralizing buffer (0.4 M Tris, pH7.5), followed by staining with 20 µg/ml propidium iodide (PI, Sigma-Aldrich Chemical Co.). The slides were examined under a fluorescence microscope.

### Protein extraction and Western blot analysis

Total cell lysates were lysed in an extraction buffer [25 mM Tris-Cl (pH 7.5), 250 mM NaCl, 5 mM EDTA, 1 % Nonidet P-40, 0.1 mM sodium orthovanadate, 2 μg/mL leupeptin, and 100 μg/mL phenylmethylsulfonyl fluoride]. The protein concentration in the cell lysate was determined using a detergent-compatible protein assay from Bio-Rad (Hercules, CA, USA). For Western blot analysis, equal amounts of protein (30~50 μg) were separated by 8~10 % sodium dodecyl sulfate (SDS)-polyacrylamide gel electrophoresis and then electro-transferred to polyvinylidene fluoride (PVDF) membranes (Schleicher & Schuell, Keene, NH, USA). The membranes were blocked with 5 % skim milk for 1 h and then subjected to immunoblot analysis with the appropriate antibodies. Using an enhanced chemiluminescence (ECL) detection system (Amersham Co., Arlington Heights, IL, USA), immunoreactive bands were detected and exposed to X-ray film. Various primary and secondary antibodies for Western blot analysis were obtained from Cell Signaling Technology, Inc. (Boston, MA, USA) and Santa Cruz Biotechnology (Santa Cruz, CA, USA). 

### Measurement of Mitochondrial Membrane Potential (MMP, Δψm)

MMP values were determined using the dual-emission potential-sensitive probe 6,6'-tetrachloro-1,1',3,3'-tetraethylimidacarbocya-nine iodide (JC-1, Sigma-Aldrich Chemical Co.). Briefly, the cells were collected and incubated with 10 μM of JC-1 for 20 min at 37 °C in the dark. After JC-1 was removed, the cells were washed with PBS to remove unbound dye, and the amount of JC-1 retained by 10,000 cells per sample was measured using a flow cytometer (Chun et al., 2016[12]).

### DNA fragmentation assay

For the extraction of fragmented DNAs, the cells were lysed in a buffer [10 mM Tris-HCl (pH 7.4), 150 mM NaCl, 5 mM EDTA, and 0.5 % Triton X-100] for 1 h at room temperature. After centrifugation at 14,000 rpm for 30 min, the supernatant was collected and incubated with proteinase K (Invitrogen, Carlsbad, CA, USA) for 3 h at 50 °C. The fragmented DNA in the supernatant was separated through 1 % agarose gel. DNA fragmentation pattern was visualized by ultraviolet light source after ethidium bromide (EtBr, Sigma-Aldrich Chemical Co.) staining. 

### Nuclear staining with DAPI

The cells were harvested, washed with PBS, and fixed with 3.7 % paraformaldehyde (Sigma-Aldrich Chemical Co.) for 30 min at room temperature. After washing with PBS twice, cells were stained with 2.5 μg/ml 4,6-dianmidino-2-phenylindole (DAPI, Sigma-Aldrich Chemical Co.) solution for 20 min at room temperature. The stained cells were washed three times with PBS and analyzed using a fluorescence microscope (Carl Zeiss, Deisenhofen, Germany).

### Determination of apoptotic cells by flow cytometry 

A fluorescein-conjugated annexin V (annexin V-FITC) staining assay kit (BD Biosciences, San Jose, CA, USA)) was used to quantitatively assess the level of induced cell apoptosis. Briefly, the cells were washed with PBS and stained with 5 μl of Annexin V-FITC and 5 μl of propidium iodide (PI) in each sample to quantify the cell number at different stages of cell death. After 15 min incubation at room temperature in the dark, the degree of apoptosis was quantified as a percentage of the Annexin V-positive and PI-negative (Annexin V^+^/PI^−^cells) cells using a flow cytometer. 

### Statistical analysis

All experiments were conducted in triplicate, and a one-way ANOVA test (SPSS 11.5 statistical software) was used to compare the mean values of each treatment. Significant differences were determined using the Duncan's multiple range test. *P < 0.05 was considered to be significant.

## Results

### SBRE protects the HaCaT keratinocytes from H_2_O_2_-induced cytotoxicity

**Figure 1:**
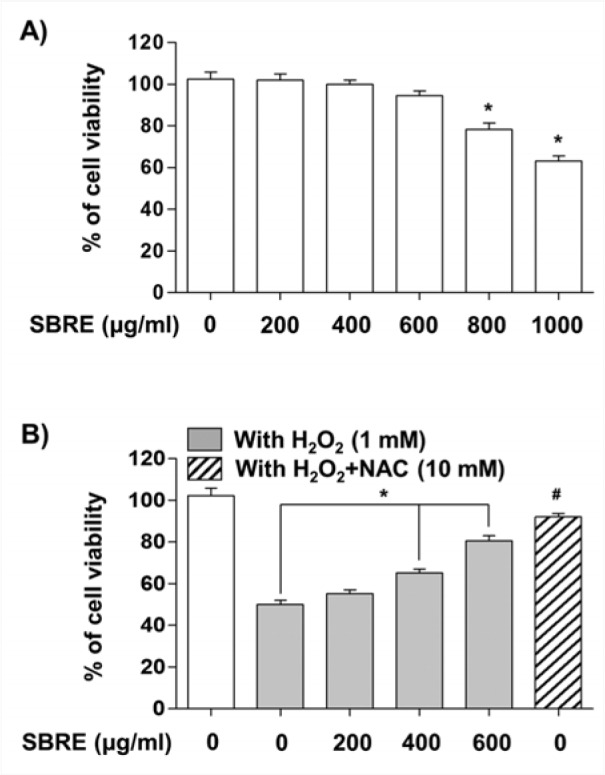
Effects of SBRE on the H_2_O_2_-induced growth inhibition in HaCaT keratinocyte cells. The cells were treated with the indicated concentrations of SBRE for 24 h (A) or pre-treated with or without SBRE (200, 400 and 600 μg/ml) and NAC (10 mM) for 1 h, then cultured with H_2_O_2 _(1 mM) for 24 h (B). The cell viability was measured by MTT assay. The results are the mean ± SD values obtained in three independent experiments (*P < 0.05 significant as compared to the H_2_O_2_-treated group; ^#^P < 0.05 *vs. *H_2_O_2_-treated cells).

Prior to investigating the protective properties of SBRE against H_2_O_2_ treatment, the cytotoxic effects of SBRE were first examined on HaCaT cells. The MTT assay indicated that the treatments did not result in any cytotoxic effect up to the concentration of 600 μg/ml, whereas cell viability significantly decreased at concentrations higher than 800 μg/ml (Figure 1A(Fig. 1)). Thus, 600 μg/ml of SBRE was selected as the optimum concentration for the subsequent examination of the protective effect of SBRE on H_2_O_2_-induced cytotoxicity. Further MTT assays revealed that treatment with 1 mM H_2_O_2 _significantly reduced cell viability; however, H_2_O_2_-induced reduction of cell viability was effectively protected by pretreatment with SBRE in a concentration-dependent manner. A pretreatment with NAC, a potent antioxidant, also effectively abrogated H_2_O_2_-induced cytotoxicity (Figure 1B(Fig. 1)).

### SBRE suppresses H_2_O_2_-induced ROS generation in HaCaT keratinocytes

**Figure 2:**
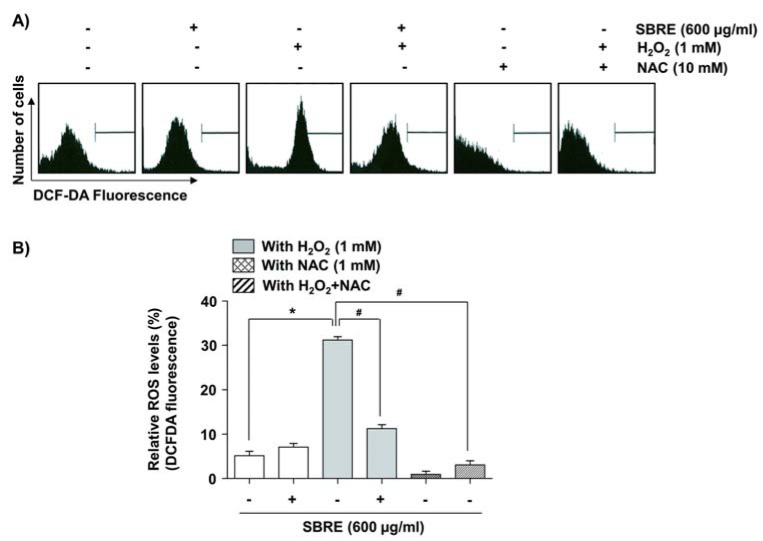
Effects of SBRE on H_2_O_2_-induced ROS generation in HaCaT keratinocyte cells. Cells were pretreated with 600 μg/ml SBRE or 10 mM NAC for 1 h and then stimulated with or without 1 mM H_2_O_2 _for 30 min. The cells were incubated at 37 °C in the dark for 20 min with culture medium containing 10 μM DCF-DA to monitor ROS production. (A) ROS generation was measured by flow cytometry. (B) Each point represents the mean ± SD of three independent experiments (*P < 0.05 *vs. *untreated control; ^#^P < 0.05 *vs. *H_2_O_2_-treated cells).

To study the mechanisms underlying the protective effect of SBRE, the radical-scavenging effect of SBRE was determined through the use of an ROS-sensitive fluorescent dye, DCF-DA. The results of the flow cytometry analysis demonstrated that HaCaT cells exposed to H_2_O_2_ for 30 min showed a significant increase in the generation of ROS, whereas this induction was substantially inhibited by SBRE or NAC pretreatment (Figure 2(Fig. 2)). The results indicated that the protective effects of SBRE against H_2_O_2_ in HaCaT cells were a direct consequence of the inhibition of excessive ROS production. 

### SBRE inhibits H_2_O_2_-induced DNA damage in HaCaT keratinocytes

**Figure 3:**
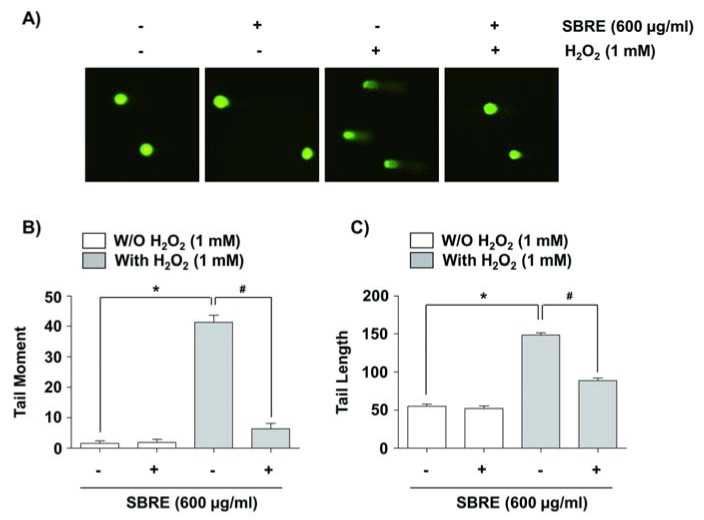
Protection of H_2_O_2_-induced DNA damage by SBRE in HaCaT keratinocyte cells. Cells were pretreated with 600 μg/ml SBRE for 1 h and then incubated with or without 1 mM H_2_O_2 _for 24 h. (A) To detect cellular DNA damage, the comet assay was performed, and representative pictures of the comets were taken using a fluorescence microscope (original magnification, 400×). (B and C) The average of the Tail Moment (TM) and Tail Length of at least 100 cells per experimental point are shown. (^*^P < 0.05 significant as compared to untreated control group; ^#^P < 0.05 significant as compared to H_2_O_2_-treated group).

**Figure 4:**
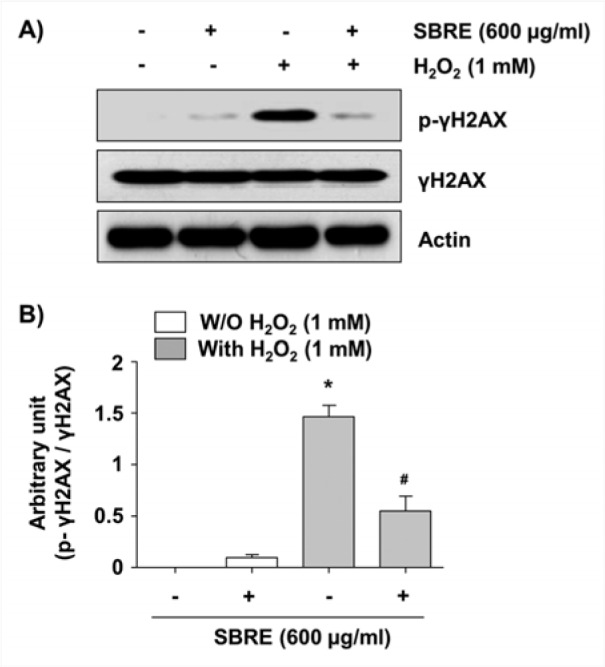
Attenuation of H_2_O_2_-induced phosphorylation of γH2AX by SBRE in HaCaT keratinocyte cells. Cells were pretreated with 600 μg/ml SBRE for 1 h and then stimulated with or without 1 mM μM H_2_O_2 _for 24 h. (A) The cells were lysed and then equal amounts of cell lysates were separated on SDS-polyacrylamide gels and transferred to PVDF membranes. The membranes were probed with specific antibodies against γH2AX and p-γH2AX, and the proteins were visualized using an ECL detection system. Actin was used as an internal control. (B) The relative expression of p-γH2AX represents the average densitometric analyses as compared with γH2AX. Each point represents the mean ± SD of three independent experiments (*P < 0.05 *vs. *untreated control; ^#^P < 0.05 *vs. *H_2_O_2_-treated cells).

We next assessed the protective effects of SBRE against H_2_O_2_-induced DNA damages using the comet assay, which measures single as well as double-strand breaks (Azqueta et al., 2014[1]). Figure 3(Fig. 3) demonstrates that H_2_O_2_ treatment increased both the amounts of DNA in the tail (tail moment) and distance of DNA migration (tail length); however, SBRE pretreatment significantly reduced both percentages elevated by H_2_O_2_. Additionally, immunoblotting results revealed that exposure of cells to H_2_O_2_ increased the phosphorylation of histone γH2AX on serine 139, a marker of DNA double strand breaks (Rogakou et al., 1998[37]); however, this adverse effect was effectively inhibited by SBRE pretreatment (Figure 4(Fig. 4)). Our findings suggested that inhibition of excessive ROS by SBRE may contribute to the attenuation of H_2_O_2_-induced DNA damage. 

### SBRE alleviates H_2_O_2_-induced mitochondrial dysfunction in HaCaT keratinocytes

**Figure 5:**
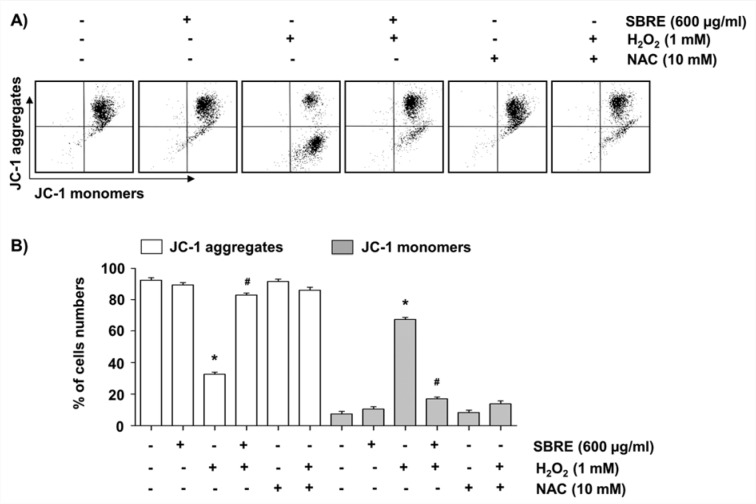
Inhibition of H_2_O_2_-induced mitochondrial dysfunction by SBRE in HaCaT keratinocyte cells. Cells were pretreated with 600 μg/ml SBRE or 10 mM NAC for 1 h and then stimulated with or without 1 mM H_2_O_2 _for 24 h. (A) The cells were collected and incubated with 10 μM JC-1 for 20 min at 37 °C in the dark. The cells were then washed once with PBS, and the values of MMP were evaluated using a flow cytometer. (B) Each point represents the mean ± SD of three independent experiments (*P < 0.05 *vs. *untreated control; ^#^P < 0.05 *vs. *H_2_O_2_-treated cells).

A change in MMP (Δ*ψ*m) was examined because mitochondria are the major sites of oxidative phosphorylation and production of ROS, and they are involved in the initiation of apoptosis through membrane permeabilization (Cha et al., 2015[6]). As shown in Figure 5(Fig. 5), H_2_O_2_-treated cells showed a loss of MMP, as substantiated by an increase in fluorescence (FL-1) with the JC-1 dye in flow cytometry analysis, indicative of mitochondrial depolarization, as compared with control cells and SBRE-treated cells. However, pretreatment with SBRE significantly blocked the loss of MMP in H_2_O_2_-treated cells as did NAC. 

### SBRE prevents H_2_O_2_-induced apoptosis in HaCaT keratinocytes

**Figure 6:**
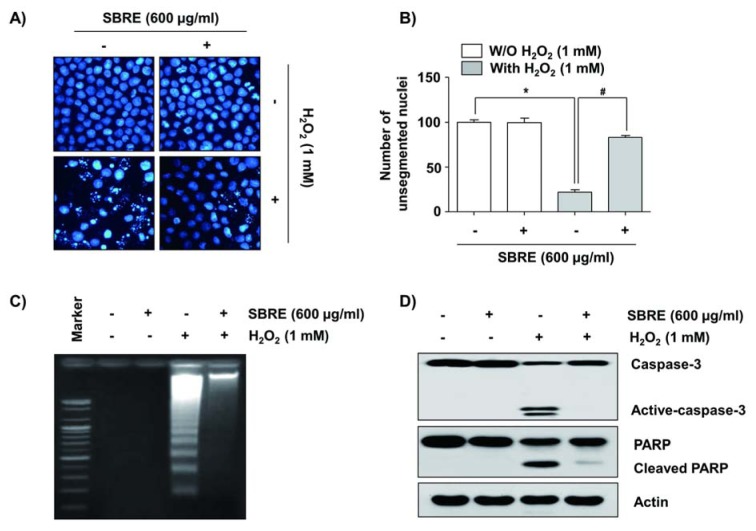
Prevention of H_2_O_2_-induced chromatin condensation, DNA fragmentation, cleavage of caspase-3 and degradation of PARP by SBRE in HaCaT keratinocyte cells. Cells were pretreated with 600 μg/ml for 1 h and then stimulated with or without 1 mM μM H_2_O_2 _for 24 h. (A and B) The cells were fixed and stained with DAPI solution. After 15 min incubation at room temperature, stained nuclei were observed using a fluorescence microscope (original magnification, 400x). Each point represents the mean ± SD of three independent experiments (*P < 0.05 *vs. *untreated control; ^#^P < 0.05 *vs. *H_2_O_2_-treated cells). (C) DNA fragmentation was analyzed by extracting the fragmented DNAs and separating them by electrophoresis in a 1.0 % agarose gel containing EtBr. (D) The cells were lysed and then equal amounts of cell lysates were separated on SDS-polyacrylamide gels and transferred to nitrocellulose membranes. The membranes were probed with specific antibodies against caspase-3 and PARP, and the proteins were visualized using an ECL detection system. Actin was used as an internal control.

**Figure 7:**
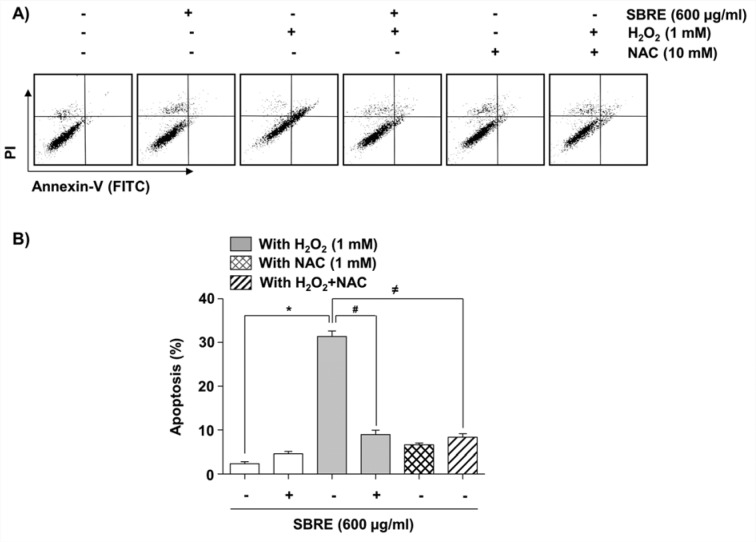
Attenuation of H_2_O_2_-induced apoptosis by SBRE in HaCaT keratinocyte cells. Cells were pretreated with 600 μg/ml SBRE or 10 mM NAC for 1 h and then stimulated with or without 1 mM H_2_O_2 _for 24 h. (A) The cells were stained with FITC-conjugated Annexin-V and PI for flow cytometry analysis. The percentages of apoptotic cells were determined by counting the percentage of Annexin V-positive cells. (B) Each point represents the mean ± SD of three independent experiments (*P < 0.05 *vs. *untreated control; ^#^P < 0.05 *vs. *H_2_O_2_-treated cells).

To elucidate the cytoprotective effect of SBRE against the H_2_O_2_-induced reduction of HaCaT cell viability, it was investigated the effects of SBRE on H_2_O_2_-mediated apoptosis. The results of nuclear morphological changes and DNA fragmentation using DAPI staining and agarose gel electrophoresis, respectively, showed that H_2_O_2_ treatment significantly increased the number of condensed or blebbing nuclei and formation of DNA laddering, whereas, when these cells were pretreated with SBRE, these phenomena were markedly reduced (Figure 6A-C(Fig. 6)). The results of flow cytometry analysis consistently indicated an increased number of annexin-positive cells, i.e., apoptotic cells, in the H_2_O_2_-treated culture compared to the untreated control group. However, the pretreatment of cells with SBRE prior to H_2_O_2 _exposure effectively protected the cells against apoptosis (Figure 7(Fig. 7)).

### SBRE attenuates H_2_O_2_-induced activation of caspase-3 and cleavage of PARP in HaCaT keratinocytes

The mitochondria-mediated apoptosis pathway is initiated by the loss of MMP and subsequent release of pro-apoptotic proteins, leading to the activation of caspase-9 and -3 (MacKenzie and Clark, 2008[32]; Hensley et al., 2013[17]). Accordingly, the active forms of caspase-3 and degradation of poly(ADP-ribose) polymerase (PARP), a well-known substrate of caspase-3 (Lazebnik et al., 1994[28]), were assessed by Western blot analysis. Our results indicated that there was a marked increase in the levels of activated caspase-3 (17 and 19 kDa) and cleaved PARP (89 kDa) expression in H_2_O_2_-treated cells compared with the control (Figure 6D(Fig. 6)). However, H_2_O_2_-induced caspase-3 activation and PARP degradation were absolutely abrogated by SBRE pretreatment, suggesting that SBRE protects HaCaT cells from apoptosis by inhibiting the mitochondrial caspase-dependent pathway. 

### SBRE enhances the expression Nrf2 and HO-1 in HaCaT keratinocytes

**Figure 8:**
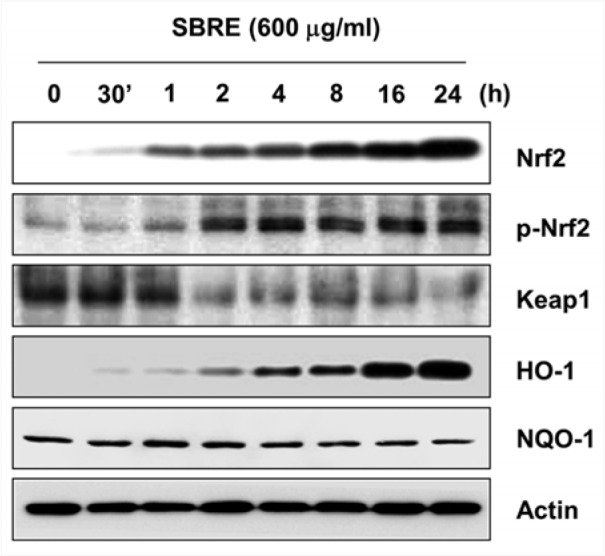
Induction of Nrf2 and HO-1 expression by SBRE in HaCaT keratinocyte cells. Cells were incubated with 600 μg/ml SBRE for the indicated periods. Total cellular proteins were separated on SDS-polyacrylamide gels and then transferred onto PVDF membranes. The membranes were probed with the indicated antibodies. Proteins were visualized using an ECL detection system. Actin was used as an internal control of total cellular and nuclear proteins.

A transcription factor Nrf2 regulates the expression of ARE-driven antioxidant and cytoprotective genes including HO-1 and NQO-1 to control the cellular defense against oxidative stress (Satoh et al., 2013[38]; Murakami and Motohashi, 2015[34]). Therefore, we further investigated whether SBRE activated Nrf2, and found that SBRE treatment gradually increased Nrf2 expression as well as its phosphorylation in a time-dependent manner, and concomitantly decreased Kelch-like ECH-associated protein 1 (Keap1) levels, a negative regulator of Nrf2 (Figure 8(Fig. 8)). Additionally, SBRE also enhanced HO-1 expression, but not NQO-1 expression, indicating that the cytoprotective effects of SBRE against oxidative stress in HaCaT cells are probably associated with activation of the Nrf2/HO-1 signaling pathway.

## Discussion

In the present study, we evaluated the antioxidant capacity of SBRE, an ethanol extract of the root of *S. baicalensis*, to prevent oxidative stress-induced cellular damage in HaCaT human skin keratinocytes. Our data from the MTT assay and flow cytometry demonstrated that SBRE significantly rescued cell viability and reduced apoptosis from H_2_O_2_-treated HaCaT cells. The protection against oxidative stress was mediated by the inhibition of DNA damage, mitochondrial dysfunction, ROS generation and caspase-3 activation. This protective effect of SBRE was also associated with the activation of the Nrf2/HO-1 signaling pathway. 

Oxidative stress, represented as an increase in ROS, is an abnormal phenomenon characterized by a production of free radicals that exceeds the antioxidant capacity. The extremely elevated levels of ROS can destroy cytoprotective defense mechanisms by the neutralizing of antioxidants, leading to the acceleration of skin aging and development of various skin diseases (Kammeyer and Luiten, 2015[21]; Karran and Brem, 2016[22]). Therefore, prevention of ROS accumulation with antioxidant agents is often regarded as a possible strategy for the management of such oxidative damage in the skin (Oresajo et al., 2012[36]; Tundis et al., 2015[45]). The generation of excess ROS can irreversibly cause a range of base modification and DNA strand breaks, resulting in DNA damage (Birch-Machin et al., 2013[2]; Katiyar, 2016[24]). Elevated levels of ROS are associated with the first events in the apoptosis by inducing mitochondrial dysfunction, resulting in the decrease of MMP, and thus activate caspase-9 and -3 through release of mitochondrial apoptotic factors into cytoplasm, which are known to be strong stimulators of the intrinsic apoptosis pathway involving the mitochondrial cascade (Brunelle and Chandel, 2002[4]; Cadet et al., 2015[5]). The activation of caspase-3 participates in the cleavage or degradation of various important proteins including PARP, which are involved in apoptosis, and eventually in the activation of other caspases. Thus, activation of caspase-3 is associated with the cleavage of PARP and has a primary role in triggering the cascade of events leading to the apoptosis pathway (MacKenzie and Clark, 2008[32]; Hensley et al., 2013[17]). In the present study, we demonstrated that SBRE significantly protected the HaCaT skin keratinocyte cells against H_2_O_2_-induced growth inhibition and DNA damage, suggesting that SBRE may potentially enhance DNA repair. Additionally, along with elevated ROS and mitochondrial dysfunction, we found the loss of MMP, activation of caspase-3 and degradation of PARP in HaCaT cells by H_2_O_2_ treatment. However, the results of JC-1 staining and immunoblotting showed SBRE could effectively restore the H_2_O_2_-induced loss of MMP to the basal level and prevent activation of caspase-3 as well as cleavage of PARP by H_2_O_2_ in HaCaT cells. These results pointed out that the ability of SBRE to attenuate oxidative stress partly depends on inhibiting mitochondrial-related apoptosis. We also found that elevated ROS accumulation in H_2_O_2_-exposed cells was substantially inhibited by SBRE pretreatment, indicating the free radical scavenging activity and protective properties of SBRE. 

Accumulating evidence strongly suggests that the Nrf2-mediated signaling pathway is essential in protecting human skin fibroblasts against oxidative stress (Satoh et al., 2013[38]; Loboda et al., 2016[31]). Under basal conditions, Nrf2-dependent transcription is suppressed by Keap1, a negative regulator of Nrf2, which facilitates the degradation of Nrf2 through ubiquitin-mediated proteasomal degradation (O'Connell et al., 2015[35]; Jaramillo and Zhang, 2013[20]). Upon modification of specific thiols by insult, Keap1 triggers the dissociation of Nrf2 from the Nrf2-Keap1 complex in the cytoplasm and allows Nrf2 to translocate to the nucleus, where it subsequently activates AREs present in the promoter regions of an array of genes (Satoh et al., 2013[38]; Murakami and Motohashi, 2015[34]). Therefore, the status of Nrf2 and its inhibitory protein Keap-1 determines the Nrf2-mediated ARE activity (Suzuki and Yamamoto, 2015[44]; Loboda et al., 2016[31]). Moreover, several lines of studies revealed that phosphorylation of Nrf2 (Try568) leads to the nuclear export of Nrf2 (Jain and Jaiswal, 2006[19]; Kaspar and Jaiswal, 2011[23]). Therefore, we investigated whether Nrf2 pathway contributes to the protective effects of SBRE against H_2_O_2_-induced oxidative stress in HaCaT cells. Our data strongly supports the ability of SBRE to stimulate the Nrf2 pathway, as SBRE treatment time-dependently increased Nrf2 accumulation and phosphorylation, and decreased Keap1 expression. Following the treatment of HaCaT cells with SBRE, we also observed a marked increase in HO-1 expression, but NQO-1 was unaffected by SBRE treatment. HO-1 is a Nrf2 downstream target and powerful indirect antioxidant enzyme, which is a rate-limiting enzyme in heme catabolism (Srisook et al., 2005[43]; Chau, 2015[8]). This enzyme converts heme to beneficial byproducts such as carbon monoxide and bilirubin that can directly scavenge free radicals and repair DNA damage caused by oxidative stress (Jaramillo and Zhang, 2013[20]; Chau, 2015[8]). Although the detailed molecular mechanism of SBRE-mediated Nrf2 activation was not yet fully established, our observations support the proposal that the cytoprotective effect of SBRE against oxidative stress in HaCaT cells is probably mediated through activating the Nrf2/HO-1 signaling pathway. Additionally, all the effects of SBRE were observed at nontoxic concentrations. Therefore SBRE could be considered as important natural active principles to include among skin photoprotective agents with promising applications in dermatological clinical research.

In conclusion, SBRE is a potent antioxidant, able to prevent oxidative DNA damage and to reduce ROS production and activation of the mitochondria-mediated apoptotic pathway in H_2_O_2_-treated HaCaT human skin keratinocyte cells. This process is also associated with the involvement of Nrf2 activation and the up-regulation of the expression of its downstream antioxidant gene HO-1 in order to protect cells from oxidative stress. Therefore, SBRE may be of therapeutic value in the prevention and treatment of various human skin diseases associated with oxidative stress. However, further studies especially those using human systems are needed to determine cellular uptake, distribution and long-term effects of SBRE on the skin for optimal protection.

## Conflicts of interest

The authors declare that there is no conflict of interest.

## Acknowledgement

This research was financially supported by the Ministry of Trade, Industry, and Energy (MOTIE), Korea, under the “Regional Specialized Industry Development Program” supervised by the Korea Institute for Advancement of Technology (KIAT, R0003671 and R0005567).
